# Risk of Fungal Infection to Dental Patients

**DOI:** 10.1155/2017/2982478

**Published:** 2017-06-14

**Authors:** Jaqueline Lopes Damasceno, Rafael Aparecido dos Santos, Amir Horiquini Barbosa, Luciana Assirati Casemiro, Regina Helena Pires, Carlos Henrique Gomes Martins

**Affiliations:** Nucleus of Research in Exact and Technological Sciences, Laboratory of Research in Applied Microbiology, University of Franca, Avenida Dr. Armando Salles de Oliveira, 201 Parque Universitário, 14404-600 Franca, SP, Brazil

## Abstract

Fungi can cause various diseases, and some pathogenic fungi have been detected in the water of dental equipment. This environment offers suitable conditions for fungal biofilms to emerge, which can facilitate mycological contamination. This study verified whether the water employed in the dental units of two dental clinics at the University of Franca was contaminated with fungi. This study also evaluated the ability of the detected fungi to form biofilms. The high-revving engine contained the largest average amount of fungi, 14.93 ± 18.18 CFU/mL. The main fungal species verified in this equipment belonged to the genera* Aspergillus* spp.,* Fusarium* spp.,* Candida* spp., and* Rhodotorula* spp. Among the isolated filamentous fungi, only one fungus of the genus* Fusarium* spp. did not form biofilms. As for yeasts, all the* Candida* spp. isolates grew as biofilm, but none of the* Rhodotorula* spp. isolates demonstrated this ability. Given that professionals and patients are often exposed to water and aerosols generated by the dental procedure, the several fungal species detected herein represent a potential risk especially to immunocompromised patients undergoing dental treatment. Therefore, frequent microbiological monitoring of the water employed in dental equipment is crucial to reduce the presence of contaminants.

## 1. Introduction

Monitoring the quality of the water employed in dental units is crucial: patients and oral care professionals are regularly exposed to water and aerosols generated in the dental unit, which can be a potential source of contamination with opportunistic organisms especially in the case of immunocompromised patients. In this sense, professionals working in the area of dentistry have concentrated efforts on developing biosecurity standards as a prophylactic measure to prevent infections from spreading in dental clinics [1]. The water that circulates in distribution networks is far from being a pure and stable product. In fact, the density of microorganisms in this water increases with the residence time and the distance between the water treatment station and the final consumer [2]. Hence, stagnant water, production of biofilm, and lack of disinfection may help microorganisms to proliferate in the water systems of dental units [3].

Microbes may grow as biofilms in the channels of the water distribution network. Microbial biofilms provide numerous pathogenic microorganisms with the conditions to survive, thereby deteriorating the quality of the distributed water [4]. In addition, fungal spores and hyphal fragments, which can act as allergens and irritants, may be aerosolized in the environment when the contaminated water passes through dental equipment [5].

Among the infections caused by opportunistic fungi, the infections caused by* Aspergillus* spp.,* Fusarium* spp., and* Acremonium* spp. stand out. As for the infections caused by non-*Candida* and non-*Cryptococcus* yeasts, the infections caused by* Trichosporon* spp.,* Malassezia* spp., and* Rhodotorula* spp. [6, 7] are worthy of note.* Candida* and* Cryptococcus* yeast species are mainly involved in the etiology of mycotic infections. In turn, candidiasis is the commonest fungal infection and may be caused by different* Candida* spp. species [8].

Bacteria, fungi, and protozoa may find favorable conditions to thrive in dental units. Literature papers have reported microorganism counts ranging from 100 to 400.000 CFU/mL in dental units [9, 10]. According to the literature, the main sources of water contamination are the city municipal water and the saliva of patients arising from sucking devices devoid of antireflux valves. Additionally, the development of biofilms in the water system of dental units is a major source of indirect contamination [4].

There are few literature studies on the fungal contamination of water in dental units and on the possible formation of biofilms [11, 12]. These issues are a reality in the routine of dental clinics and are worth monitoring if patients are to be successfully treated. This study has investigated and quantified the presence of yeast and filamentous fungi in the water of two dental clinics, the Surgery and the Periodontics clinics, of the Dentistry College of the University of Franca. We have isolated the microorganisms and assessed the* in vitro* sensitivity of the yeast fungi to fluconazole. We have also analyzed the ability of the microorganisms to form biofilms, and we have determined the time elapsed until biofilms of the main fungal genera isolated herein emerged.

## 2. Materials and Methods

### 2.1. Sampling

The water samples were collected at specific points of two dental clinics of the Dentistry College of the University of Franca (UNIFRAN) along approximately nine months. The collection points were the central water reservoir that supplies the clinics (CR), the water reservoirs of the dental units (RU), the water of the triple syringes (TS), and the water of the high-revving engine (HR).

Briefly, the water samples were collected at four points (as specified above CR, RU, TS, and HR) of five dental units (1 to 5) located in two clinics (the surgical clinic (CC) and the periodontics clinic (CP)), in duplicate (A and B). Three series of samples were collected. The first series consisted of the standardized water supply at UNIFRAN (deionized water), the second series consisted of sterile water, and the third series consisted of the standardized water supply at UNIFRAN (deionized water) collected after the plastic connections of the equipment had been exchanged for new connections. The total number of samples was 240. These samples were placed in 100-mL sterile vials. The time elapsed between sample collection and the beginning of sample processing was approximately 30 min.

### 2.2. Sample Processing and Microorganism Counting

The samples were aseptically collected after allowing the water to flow for 2 min. The water samples were filtered through a cellulose acetate membrane (0.45-*μ*m pore size, Millipore, SP, Brazil) on a filtration ramp (Sartorius do Brasil Ltda). The membrane was placed on a plate containing R2A agar (Difco, Detroit, MI, USA) at 30°C for 7 to 14 days, to allow filamentous fungi and yeasts to grow. After the incubation period, the colonies were counted, and the results were expressed as CFU/mL. One of the colonies with the same morphology within the same board was subcultured for identification in Sabouraud Dextrose plus chloramphenicol.

### 2.3. Identification of Yeast Isolates

To identify the microorganisms, the species were defined on the basis of the colony morphology, the results of the germ tube test, the results of the urease test, the presence of chlamydospores on Tween 80 [13–15] meal agar (Difco), the carbohydrate fermentation tests (zymogram), and the pattern of assimilation of a variety of carbon and nitrogen sources (auxanogram).* CHROMagar Candida* aided the initial screening and helped to confirm the purity of the isolates [16]. The crops were identified according to the keys described by Kurtzman & Fell [17].

### 2.4. Susceptibility Tests on Yeasts

To standardize the inoculum, the yeast strains were seeded in Sabouraud Dextrose agar (Difco) and incubated in an oven at 25°C for 24 h. The colonies were suspended in 5.0 mL of 0.85% saline and homogenized. The cells were counted on a Neubauer chamber. When necessary, the suspensions were diluted to obtain 1 × 10^4^ cells/mL.

Susceptibility to fluconazole was assayed by the disc diffusion test. The minimum inhibitory concentration data were interpreted as described in the criteria of the Clinical and Laboratory Standards Institute [18], document M44-A2. Fluconazole (25 *µ*g) discs were obtained from CECON Inc. (Centro de Controle e Produtos para Diagnósticos Ltda, São Paulo, Brazil, Batch Number 1786). To conduct the disc diffusion tests, plates containing Mueller-Hinton agar (Difco) supplemented with 2% glucose and methylene blue (0–5 *µ*g/mL) were used. The plates were incubated at 35–37°C in air and read at 24–48 h. The* Candida krusei* ATTC 6258 and* Candida albicans* ATCC 90028 strains, which are resistant and susceptible to fluconazole, respectively, were used as quality controls.

### 2.5. Identification of the Isolated Filamentous Fungi

After being cultured on potato dextrose agar (Difco), the genera of all the filamentous fungi were identified by traditional methods, on the basis of their macromorphology and microscopic structures [19–21].

### 2.6. *In Vitro* Biofilm Assay

The protocol for biofilm growth described by Pierce et al. [22] was used. Briefly, the biofilms were formed on commercially available, presterilized polystyrene flat-bottom 96-well microtitre plates (Corning Inc., Corning, NY, USA). Standard cell suspensions (100 *μ*L of a suspension containing 1 × 10^6^ cells/mL in Roswell Park Memorial Institute (RPMI) 1640 supplemented with L-glutamine and buffered with 0.165 M morpholinepropanesulfonic acid (MOPS, from Sigma)) were seeded in selected wells and incubated at 37°C for 40 h.

The yeasts were subcultured for 18–20 h and quantified. Then, dilutions containing 2 × 10^6^ cells/mL were prepared. Aliquots of 100 *µ*L were inoculated in 96-well microtitre plates and incubated at 37°C for 48 h. As for the filamentous fungi, the cells were quantified, and dilutions containing 2 × 10^5^ cells/mL were prepared. Aliquots of 200 *µ*L were inoculated into 96-well microtitre plates and incubated at 37°C for 72 h.

The resulting biofilms were semiquantitatively measured by the 2,3-bis(2-methoxy-4-nitro-5-sulfophenyl)-2-H-tetrazolium-5-carboxanilide (MTT, from Sigma) reduction assay. To this end, an MTT solution (20 *μ*L) (a stock solution containing 5 mg of MTT per mL of phosphate buffered saline [PBS]) was added to each well, and the samples were incubated at 37°C for 3 h, in the dark. After the incubation period, the plates were washed three times with PBS. Isopropyl alcohol (200 *μ*L) was added, to solubilize the MTT formazan product. Then, 100 *μ*L of this solution was transferred to a new microtitre plate. The optical density (OD) of the formazan present in the destaining solution was measured at 540 nm with a microplate reader (Asys, Eugendorf, Salzburg, Austria). An optical density of 0.5 confirmed formation of biofilm [22, 23].

### 2.7. Scanning Electron Microscopy (SEM)

To conduct SEM, the biofilms were grown on sterile PVC discs placed in 24-well microtitre plates (Corning). To this end, 300 *µ*L of standardized cell suspensions containing 2.5 × 10^6^ cells/mL in RPMI 1640 were placed on the appropriate discs at 37°C [24]. The biofilms were assayed as previously described by Priester et al. [25] and Pires et al. [26]. After processing, the samples were observed under a scanning electron microscope (JEOL, model JSM 5410, Japan) operating in the high-vacuum mode, at 15 kV. The images were processed for display with the aid of the Photoshop software (Adobe Systems Inc., Mountain View, CA.).

## 3. Results

### 3.1. Quantitative Analysis of Water Samples Collected from Dental Equipment

The number of fungal colonies at the various collection points ranged from 0 to 40 CFU/mL. The mean and the standard deviation (in CFU/mL) of the counts were as follows: 6.80 ± 11.14, 4.20 ± 7.67, 4.00 ± 7.44, and 14.93 ± 18.18 in the central water reservoir that supplies the clinics (CR), in the water reservoirs of the dental units (RU), in the water of the triple syringes (TS), and in the water of the high-revving engine (HR), respectively.

The percent distribution of the fungal isolates in the samples collected at different points of the dental units, namely, HR, TS, RU, and CR, was as follows:* Aspergillus* spp.: 38, 13, 11, and 5, respectively;* Fusarium* spp.: 27, 27, 46, and 14, respectively;* Penicillium* spp.: 5, 16, 11, and 48, respectively;* Rhodotorula minuta*: 2, 3, 3, and 19, respectively;* Rhodotorula rubra*: 14, 16, 13, and 9, respectively;* Candida lusitaniae*: 5, 3, 8, and 0, respectively; and* Candida guilliermondii*: 9, 16, 7, and 0, respectively. The percent distribution of fungi of the genera* Trichoderma* spp.,* Acremonium* spp.,* Bipolaris* spp., and* Rhinocladiella* spp. was 1, 3, 5, and 3 in RU, TS, CR, and TS, respectively.

### 3.2. Quantification and Visualization of the Biofilms

**Figure 1:**
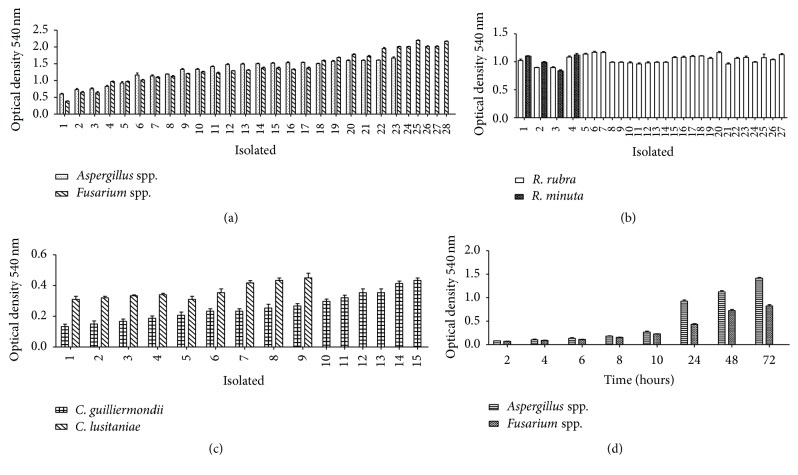
(a) Formation of biofilms consisting of* Aspergillus* spp. and* Fusarium *spp. isolates after 72 h of incubation; (b) formation of biofilms consisting of* R. rubra* and* R. minuta* isolates after 72 h of incubation; (c) formation of biofilms consisting of* C. guilliermondii* and* C. lusitaniae* isolates after 24 h of incubation; (d) kinetics of the formation of biofilms consisting of the filamentous fungi* Aspergillus* spp. and* Fusarium *spp. after 72 h of incubation.

A wide range of microbial species can grow as biofilm. Here, we tested biofilm growth for 72 h in the case of* Aspergillus* spp. and* Fusarium* spp. (Figure 1(a)) and of* R. rubra* and* R. minuta* (Figure 1(b)) isolates and for 24 h in the case of* C. guilliermondii* and* C. lusitaniae* (Figure 1(c)) yeasts. Cell viability was confirmed by optical density readings. Note the variability among the different isolates, represented by each column of the graphs. Among the filamentous fungi, only isolate number 1 of* Fusarium* spp. did not form biofilms after 72 h of incubation. Among the yeast isolates, biofilm formation was not significant during the incubation period (OD < 0.5).

**Figure 2:**
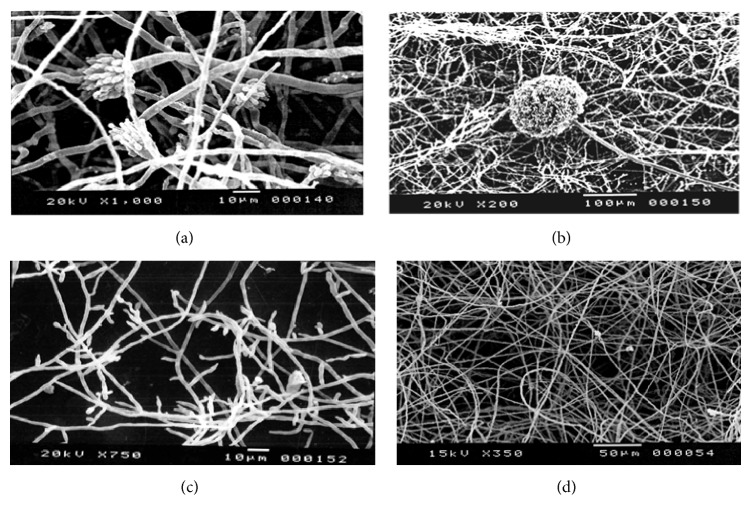
Photomicrographs of the biofilms consisting of* Aspergillus* sp. at magnifications of 10 *µ*m (a) and 100 *µ*m (b) and of biofilms consisting of* Fusarium* sp. at magnifications of 10 *µ*m (c) and 50 *µ*m (d).

In this study, the mature biofilms consisting of* Aspergillus* (Figures 2(a) and 2(b)) and* Fusarium* (Figures 2(c) and 2(d)) species displayed hyphae and conidial structures, as demonstrated by SEM, which evidenced that these fungi had the ability to form biofilms.

The kinetics of biofilm formation of the filamentous fungal isolates of* Aspergillus* spp. and* Fusarium* spp. presented in Figure 1(d) revealed significant accumulation of viable cells, expressed in OD, at 10 h of incubation. The OD stabilized between 24 and 48 h, reflecting the maturity of the biofilm.

### 3.3. Identification and Sensitivity of the Isolated Yeasts

Macro- and micromorphological analyses helped to identify 55 yeast isolates. On the basis of the biochemical and physiological profile, 15, 9, 27, and 4 strains were compatible with* Candida guilliermondii*,* C. lusitaniae*,* Rhodotorula rubra*, and* R. minuta*, respectively.

As for the susceptibility of the yeasts to fluconazole, the* R. rubra* and* R. minuta* strains were 100% resistant to this drug, whereas the* C. guilliermondii* and* C. lusitaniae* strains were approximately 80% sensitive to fluconazole.

## 4. Discussion

Since the late 1970s, fungal infections have become a significant cause of morbidity and mortality [27]. Water running through the tubes of dental units is known to contain multiple microorganisms. This water is a potential source of microbial contamination by aerosol and hence a potential threat to the patients' and professionals' health. The American Dental Association has established that the bacterial load in the water of dental units must not exceed 200 CFU/mL, but a limit of fungal load has not been mentioned [28].

The samples assayed herein had distinct fungal loads. The level of contamination depends on parameters such as the internal diameter of the tubing, the internal surface area-to-volume ratio, the water pressure, the flow rate, and the stagnation periods. Therefore, the water tubing of dental units constitutes a source of waterborne opportunistic pathogens because the tubing offers an optimal environment for sessile microbiological communities to develop [29].

In this study, a water sample collected from the high-revving engine scored the highest in terms of fungal colonies 40 CFU/mL (Table 1 [SCRC2]). In a similar study, Szymanska [30] showed that the level of contamination with fungal species was two times higher in the high-revving engine as compared to the water reservoir.

This study identified the following microorganisms:* Aspergillus* spp.,* Fusarium* spp.,* R. rubra*,* R. minuta*,* C. guilliermondii*, and* C. lusitaniae*. Oral candidiasis is the commonest fungal infection in humans [8]. During dental treatment, direct contact with water contaminated with fungi such as* Candida* and* Aspergillus* may cause various respiratory infections (such as asthma), allergies, and wounds on mucosal membranes especially in the case of immunocompromised patients [31].* Fusarium* species are the second commonest mould causing invasive fungal infections in immunocompromised individuals. Aspergillosis has been reported as the second most prevalent opportunistic fungal infection.* R. minuta* and* R. rubra* belong to the genus* Rhodotorula*, which is related to diseases such as endocarditis, fungemia, and nonhealing oral ulcers, whereas white patches are the reported oral presentations of infection due to* Rhodotorula* [32–34].

Lisboa et al. [11] also evaluated the quality of water in dental units of the public dental care system in Maceió, Alagoas, Brazil, to find 212 isolates and 16 genera of filamentous fungi, including* Fusarium* spp. and* Aspergillus* spp. A recent study assessed fungal colonization in 41 dental units in Istanbul, Turkey. Nonsporulating fungi were detected in seven units.* Aspergillus*,* Penicillium*,* Cryptococcus*, and even* C. guilliermondii* were some of the filamentous fungi and yeasts identified in the samples. The average fungal contamination ranged from 10 to 101 CFU/mL [31].

Comparing the three series of samplings analyzed herein, the use of sterile water clearly reduced contamination (Table 1). Lizon et al. [35] detected biofilm and opportunistic microorganisms in their study about the quality of water in dental units. These authors showed that treatment with continuous disinfection associated with the use of sterile water restored the quality of water at the output of the dental care units and ensured the safety of care. According to Pankhurst et al. [36], sterile water must be used in any surgical treatment.

The high surface-volume ratio, the laminar flow, and the frequent stagnation periods of the waterlines of dental units facilitate the attachment of microorganisms and the development of biofilm. These phenomena continuously contaminate water at the outlet of dental units. This contamination may underlie the potential risk of infection due to exposure to droplets or splashed water or inhalation of aerosolized water [37]. Biofilm arises where the solid surface is in contact with water for a certain period of time. Biofilms are defined as an association of microbial cells, attached to biotic or abiotic surfaces, surrounded by a complex matrix consisting of extracellular polymeric substances [38].

Cobb et al. [39] evaluated how time-dependent waterline flushing affected the presence of biofilm. They also examined the waterlines of the dental units by scanning electron microscopy, to confirm that a residual biofilm existed in the lumen of the waterline of each dental unit. These authors concluded that it was possible to reduce planktonic bacteria significantly by continuously flushing the water of the dental unit (as analyzed at 1, 2, 3, and 4 min after the start of flushing) as compared to the baseline. However, the bacterial load (in CFU) measured after four minutes of continuous water flushing still exceeded the current ADA recommendations for acceptable levels of microorganisms.

Filamentous fungi can also be considered as biofilm-forming organisms because they are well adapted to grow on both biotic and abiotic surfaces. The formation of biofilms consisting of filamentous fungi has rarely been described.* Aspergillus niger* has been cultivated on polyester fabric, and* Fusarium solani* and* F. oxysporum* have been analyzed in contact lens [40–42]. Here, SEM studies attested that the biofilms consisting of* Aspergillus* (Figures 2(a) and 2(b)) and* Fusarium* (Figures 2(c) and 2(d)) were mature: the images revealed a dense mass of hyphae and conidial structures, which corroborated with the previous observations that filamentous fungi can grow as biofilm. This is the first study that has demonstrated the presence of fungal isolates in water used for dental purposes.

A recent study has evaluated sixteen samples of water from the pipes of dental units, to find that five* Candida guilliermondii* strains were detected. In the planktonic form, this species was susceptible to antifungal agents; however, the mature biofilm of this species was resistant to antifungal drugs [12]. Souza-Gugelmin et al. [43] demonstrated microbial contamination of the waterline of dental equipment due to formation of microbial biofilm. These authors found that the level of contamination of the water samples collected from both the high-revving engine and the triple syringe was significantly higher than the initial level of contamination verified in the water reservoir. Here, the high-revving engine presented the highest level of contamination, followed by the reservoir that supplies the clinics. Although most of the microorganisms detected in this study were able to form biofilms, biofilm formation may not have occurred because the dental professionals abode by the specifications concerning equipment cleaning and care.

Fluconazole is preferentially used to treat candidemia. Here, most* C. lusitaniae* and* C. guilliermondii* isolates were sensitive to this antifungal in the diffusion disc test [44]. All the* Rhodotorula* spp. strains were resistant to fluconazole. Diekema et al. [45] tested* Rhodotorula* strains obtained between 1987 and 2003 against eight antifungals. The strains exhibited* in vitro* resistance to fluconazole, which is an important property provided that the drug be selected to treat nosocomial fungal infections. Non-*Candida albicans* species lack many of the virulence factors present in the virulent strains. They have low ability to adhere to surfaces and secrete fewer proteinases, which may explain the inability of the microorganisms isolated here to form biofilms [8].

The guidelines of the Centers for Disease Control and Prevention (CDC) issued in 2003 advocated that the total viable count (TVT, various cut-offs) in nonsurgical dental procedures must be no higher than 500 CFU/mL [28, 46]. Given this parameter, the samples assayed herein displayed low level of contamination. However, even if the total microbial count does not always represent a risk to patients and health care workers, the presence of an opportunistic pathogen may be dangerous, especially when it is associated with other microorganisms that prefer water habitats [3]. Despite the many studies concerning the high levels of bacterial contamination in water of dental units, little is known about fungal contamination in these units. The findings of the present investigation stress the need to perform frequent microbiological monitoring of the water of dental units, to reduce the presence of contaminants in this environment.

## 5. Conclusions

This work has revealed that water used in dental equipment is contaminated with several fungal species. This contamination represents a potential risk to professionals and patients, especially immunocompromised patients undergoing dental treatment. The ability of most of the isolated fungi to form biofilms pointed out that it is difficult to eliminate these microorganisms from this environment. This is because the microorganisms function as a “community” complex, which can be the main factor underlying contamination.

## Figures and Tables

**Table 1:** Contamination level (CFU/mL) of water in five pieces of dental equipment obtained in duplicate at four collection points in two dental clinics of the University of Franca in three different sets (with deionized water, with sterile water, and with replaced plastic connections).

Number of samples	HR			TS			RU			CR		
	A	B	Total	A	B	Total	A	B	Total	A	B	Total
SCD1	2	1	3	1	0	1	1	1	2	0	0	0
SCD2	0	0	0	1	1	2	1	1	2	0	0	0
SCD3	2	2	4	0	1	1	19	1	20	1	0	1
SCD4	1	1	2	0	1	1	0	0	0	0	21	21
SCD5	0	3	3	1	20	21	1	1	2	0	20	20
PCD1	2	20	22	2	0	2	3	0	3	0	0	0
PCD2	1	5	6	1	0	1	1	8	9	0	22	22
PCD3	20	6	26	4	12	16	4	6	10	0	5	5
PCD4	21	0	21	1	0	1	1	0	1	0	0	0
PCD5	0	0	0	0	20	20	4	2	6	0	34	34
Mean			8.70			6.60			5.50			10.30
SD			10.10			8.66			6.11			12.68
SE			6.26			5.37			3.79			7.86
UL			14.96			11.97			9.29			18.16
IL			2.44			1.23			1.71			2.44

SCS1	1	0	1	3	0	3	2	0	2	0	0	0
SCS2	0	0	0	0	0	0	0	0	0	0	0	0
SCS3	0	0	0	0	0	0	0	0	0	0	0	0
SCS4	0	2	2	3	0	3	0	0	0	0	5	5
SCS5	0	32	32	0	0	0	0	1	1	0	3	3
PCS1	0	0	0	0	0	0	4	0	4	0	0	0
PCS2	0	0	0	0	0	0	0	0	0	2	0	2
PCS3	1	0	1	0	0	0	0	0	0	1	0	1
PCS4	0	2	2	0	0	0	2	0	2	0	5	5
PCS5	0	1	1	0	0	0	0	0	0	0	1	1
Mean			3.90			0.60			0.90			1.70
SD			9.90			1.26			1.37			2.00
SE			6.14			0.78			0.85			1.24
UL			10.04			1.38			1.75			2.94
IL			−2.24			−0.18			0.05			0.46

SCRC1	39	0	39	0	0	0	1	0	1	2	0	2
SCRC2	40	34	74	1	0	1	22	0	22	0	1	1
SCRC3	20	31	51	0	0	0	3	0	3	32	1	33
SCRC4	0	20	20	0	25	25	0	1	1	1	1	2
SCRC5	0	20	20	0	0	0	0	1	1	1	1	2
PCRC1	23	0	23	0	0	0	1	1	2	3	1	4
PCRC2	31	1	32	1	0	1	33	0	33	0	1	1
PCRC3	15	0	15	3	0	3	0	0	0	35	1	36
PCRC4	1	34	35	0	0	0	0	1	1	1	1	2
PCRC5	0	13	13	0	18	18	0	0	0	0	1	1
Mean			32.20			4.80			6.40			8.40
SD			18.86			9.00			11.45			13.80
SE			11.69			5.58			7.10			8.55
UL			43.89			10.38			13.50			16.95
IL			20.51			−0.78			−0.70			−0.15

SCD: Surgical Clinic with deionized water; PCD: Periodontics Clinic with deionized water; SCS: Surgical Clinic with sterile water; PCS: Periodontics Clinic with sterile water; SCRC: Surgical Clinic with replaced plastic connections; PCRC: Periodontics Clinic with replaced plastic connections; SD: standard deviation; SE: standard error; UL: upper limit confidence interval 95%; IL: lower confidence interval 95%; HR: high-revving engine; TS: triple syringes; RU: reservoirs of units (equipment); CR: central reservoir; 1 to 5 refer to the number of pieces of equipment at each collection point (five pieces of equipment for each collection point).
